# Revisiting the Role of Tolvaptan in the Management of Hyponatraemia in Acute Heart Failure: Balancing Quality of Life and Cost With Tolvaptan Treatment

**DOI:** 10.7759/cureus.93460

**Published:** 2025-09-29

**Authors:** Idowu Olaogun, Ekenechukwu E Young, Obumneme B Anyim

**Affiliations:** 1 Endocrinology, Bristol Royal Infirmary, Bristol, GBR; 2 Medicine, Stockport NHS Foundation Trust, Stockport, GBR; 3 Medicine, University of Nigeria Teaching Hospital, Enugu, NGA

**Keywords:** cost-effectiveness, guidelines, heart failure, hyponatraemia, tolvaptan

## Abstract

Hyponatraemia is a poor prognostic index in heart failure. It is a common complication of heart failure, associated with poorer outcomes including prolonged hospitalisation, increased morbidity, and reduced quality of life. For patients who have already developed this complication, the focus should be on improving their quality of life. The conventional management of hyponatraemia in heart failure largely depends on the cause and usually involves the intensification of the anti-heart failure regimen. For patients with severe hyponatraemia, it could mean an extra week's stay in the hospital on average or even longer, resulting in an increased rate of complications associated with prolonged hospitalisation. This not only increases healthcare costs but also exposes patients, particularly older adults, to risks related to immobility and hospital-acquired infections.

In theory, tolvaptan could bridge this gap in selected patients to reduce hospitalisation and improve quality of life. Tolvaptan is a selective vasopressin V2 receptor antagonist. Arginine vasopressin (AVP) plays a significant role in the pathophysiology of heart failure. It inhibits the action of AVP on renal collecting ducts, promoting free water excretion. Tolvaptan increases serum sodium and reduces diuretic doses. While it has not been associated with increased mortality, evidence on morbidity and long-term outcomes remains inconsistent. In addition, high cost and safety concerns restrict its widespread use.

This review highlights the role of tolvaptan beyond biochemical correction, with a focus on its potential to improve quality of life, reduce hospitalisations, and provide cost-effective benefits in carefully selected heart failure patients with hyponatraemia. By addressing these broader clinical and economic considerations, we provide a more balanced framework for evaluating its place in therapy. Nonetheless, further large, randomised controlled trials are required to confirm its long-term safety, cost-effectiveness, and overall impact on outcomes in this population.

## Introduction and background

Hyponatraemia is a frequent and clinically significant complication in patients with heart failure. It is associated with poor clinical outcomes, higher rates of hospitalisation, prolonged hospital stay, and an increased risk of inpatient complications [1]. The presence of hyponatraemia in heart failure patients is not merely a marker of disease severity but also a contributor to the progression of heart failure, hence further complicating its clinical management [2].

The management of hyponatraemia in heart failure is particularly challenging due to its multifactorial nature, involving neurohormonal activation, reduced renal perfusion, and fluid overload [3]. The current conventional approaches to managing hyponatraemia in heart failure include fluid restriction, optimisation of heart failure medication such as diuretics, and correction of underlying causes if identified [4]. However, these methods are limited in their ability to correct serum sodium levels in a timely, effective, and sustainable manner, leading to ongoing risks for patients [5].

The complexity of managing hyponatraemia in this population often requires advanced therapeutic strategies and goes beyond conventional treatment. As a result, there has been a growing interest in the adoption of alternative treatments that can more effectively manage hyponatraemia in this vulnerable population [6].

Tolvaptan is an oral, selective vasopressin V2 receptor antagonist used to treat euvolaemic and hypervolaemic hyponatraemia, including in heart failure patients [3]. Tolvaptan promotes aquaresis, the excretion of electrolyte-free water, without significant sodium loss, which allows for the correction of serum sodium levels. Although tolvaptan use usually avoids worsening of volume depletion, it can cause hypotension and volume depletion if not monitored carefully [4,7].

However, while tolvaptan represents a potentially valuable addition to the therapeutic arsenal for managing hyponatraemia in heart failure, its use is not without concerns. Clinical trials such as the EVEREST (Efficacy of Vasopressin Antagonism in Heart Failure: Outcome Study with Tolvaptan) trial have shown modest benefits in long-term outcomes, despite improved sodium correction [7]. Furthermore, its high cost, risk of rapid sodium overcorrection, and potential hepatotoxicity, particularly with long-term use, pose limitations to its widespread use [8,9].

This review aims to review the role of tolvaptan in improving clinical outcomes for heart failure patients with hyponatraemia. It will explore its clinical efficacy, cost-effectiveness, and potential to reduce hospitalisations.

## Review

Definition and classification of heart failure

Heart failure is a clinical syndrome with varying aetiologies, which results from impaired ventricular filling or poor ventricular contractility, hence leading to inadequate cardiac output to meet systemic metabolic demands. It is usually classified based on its effect on the calculated ejection fraction. Heart failure is thus classified into heart failure with reduced ejection fraction (HFrEF), heart failure with mid-range ejection fraction (HFmEF), and heart failure with preserved ejection fraction (HFpEF). This influences the type of treatment as well as prognosis. The most recent European and American guidelines provide detailed diagnostic and therapeutic approaches, highlighting the importance of personalised treatment strategies and the adoption of new therapeutic agents. It also emphasises the role of a multidisciplinary care approach [10,11]. These guidelines emphasise the importance of managing comorbidities, including hyponatraemia, and advocate for shared decision-making in advanced heart failure.

Pathophysiology of hyponatraemia in heart failure

The pathophysiology of heart failure involves intricate neurohormonal mechanisms, including the activation of the sympathetic nervous system, the renin-angiotensin-aldosterone system, and the release of various hormones such as antidiuretic hormone (ADH), also known as arginine vasopressin (AVP). These mechanisms initially act as compensatory responses to maintain cardiac output and blood pressure; however, over time, they contribute to the progression of heart failure. Persistent hyponatraemia typically reflects advanced heart failure and is associated with worse outcomes, including increased mortality, due to the challenges in its management. For instance, in the study by Verbalis et al. [2], the authors found that patients who achieved normal sodium levels had significantly lower mortality rates compared to those who remained hyponatraemic. Furthermore, the study suggested that the early identification and treatment of hyponatraemia in heart failure could improve overall management and outcome.

Epidemiology and classification of heart failure-associated hyponatraemia

Hyponatraemia is observed in approximately 20-30% of patients hospitalised with acute decompensated heart failure [12]. The prevalence may be higher in patients with advanced heart failure or those on diuretics, which exacerbate sodium loss [13]. Hyponatraemia in heart failure can be categorised into different forms based on its underlying mechanisms and severity. These include dilutional hyponatraemia, which is most commonly contributed to by the renin-angiotensin-aldosterone system, AVP, and other neurohormonal mechanisms and is the main driver, as well as hypovolaemic hyponatraemia mainly due to the use of diuretics. Although euvolaemic hyponatraemia from syndrome of inappropriate antidiuretic hormone secretion (SIADH) could occur, it is significantly rare [13]. Assessment should, therefore, follow a structured approach, considering clinical, biochemical, and other parameters, as this will guide treatment [13].

Clinical impact of hyponatraemia in heart failure

Length of Hospital Admission

Patients with hyponatraemia at admission or those who develop it during hospitalisation for heart failure generally have a longer hospital stay. Hyponatraemia was found to be associated with a 55% longer length of stay compared to patients with normal sodium levels. The median length of stay for patients with hyponatraemia was 11 days, compared to eight days in those who had normal sodium levels [14].

Rehospitalisation Rates

Hyponatraemia in heart failure is also linked to higher rehospitalisation rates. Patients with lower sodium levels have been shown to have a significantly higher risk of rehospitalisation within 30 days of discharge [15]. Other risk factors for rehospitalisation in this study were fluid retention and high pro-brain natriuretic peptide levels.

Mortality and Other Endpoints

Hyponatraemia is a strong predictor of both in-hospital and long-term mortality in patients with heart failure. Analysis by Gheorghiade et al. revealed that even mild hyponatraemia increased the risk of in-hospital mortality by 60% and this risk continued post-discharge, contributing to long-term mortality [12]. Additionally, worsening hyponatraemia during hospitalisation is particularly ominous, leading to a higher incidence of adverse outcomes, including the need for intensive care or mechanical support [12].

The role of AVP in the pathophysiology of hyponatraemia in heart failure

Hyponatraemia in heart failure is a common and prognostically significant electrolyte disorder. The pathophysiology of hyponatraemia in heart failure is primarily related to the inappropriate secretion of AVP in response to decreased cardiac output and reduced effective circulating volume.

Hyponatraemia in heart failure, particularly when mediated by AVP, is associated with worse clinical outcomes, including increased mortality and morbidity. This reflects the severity of the underlying neurohormonal dysregulation and fluid imbalance [12].

AVP is a key player in the neurohormonal mechanisms underlying heart failure, contributing to fluid retention and vascular resistance. While it initially acts to preserve circulatory function in the setting of reduced cardiac output, chronic elevation of AVP has adverse effects that exacerbate heart failure. Understanding the role of AVP in this context is crucial for the development of effective therapeutic strategies aimed at mitigating its negative impact. 

Under normal circumstances, AVP release is regulated by plasma osmolality; however, in heart failure, its release is predominantly driven by baroreceptors sensing reduced effective arterial volume [16].

AVP acts on the V2 receptors in the renal collecting ducts, promoting water reabsorption independent of sodium. This results in the retention of free water, which dilutes the plasma sodium concentration, leading to dilutional hyponatraemia [17]. The persistent release of AVP despite an already expanded extracellular fluid volume exacerbates this condition, as the body erroneously perceives a need to conserve water due to decreased effective circulating volume, despite actual fluid overload. The levels of AVP have been found to rise progressively with increasing New York Heart Association (NYHA) class of heart failure [18]. 

**Figure 1:**
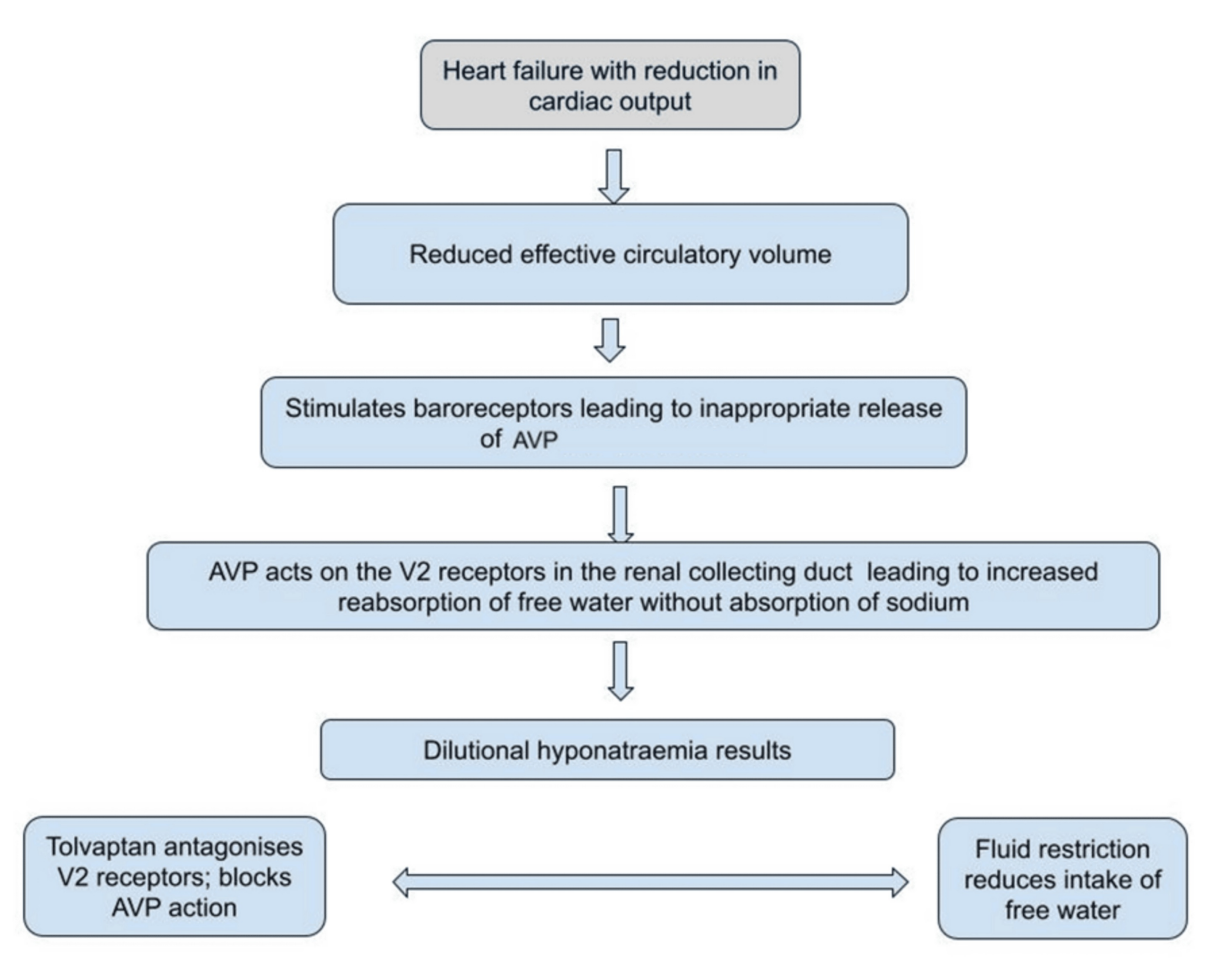
A schematic representation of the role of ADH in the pathophysiology of hyponatraemia in heart failure AVP: arginine vasopressin; ADH: antidiuretic hormone Credit: Ekenechukwu E. Young (Original image created with Google Drawings (Google LLC, Mountain View, California, United States))

The understanding of the role of AVP has led to therapeutic approaches targeting this pathway, such as the use of vasopressin receptor antagonists (vaptans) that block the action of AVP on the kidneys, thereby promoting free water excretion without losing sodium [19]. This improves some of the symptoms associated with advanced heart failure [20]. However, the clinical benefits of such treatment need to be balanced against potential risks, including the development of hypernatraemia and hypotension [21]. Figure 1 is a schematic description of the contribution of AVP to hyponatraemia in heart failure.

Natural history of hyponatraemia in heart failure

Hyponatraemia is closely linked to the progression and severity of heart failure. Early detection and management are essential, as persistent or worsening hyponatraemia often signals poor prognosis. The duration of hyponatraemia from the diagnosis of heart failure to the end of life can vary significantly, based on the severity of the disease and the patient's response to treatment.

The onset of hyponatraemia may occur early in the disease process, particularly in those with significant neurohormonal activation and fluid retention [12]. As heart failure progresses, hyponatraemia often becomes more pronounced. Persistent hyponatraemia during the chronic phase of heart failure is linked to ongoing neurohormonal activation, worsening cardiac function, and the use of diuretics. The duration of hyponatraemia in this phase can be prolonged, lasting months to years, depending on disease management and response to treatment [22].

In episodes of acute decompensated heart failure, hyponatraemia may worsen acutely. This acute phase can last from days to weeks, depending on the severity of the episode and the effectiveness of treatment. Patients often require hospitalisation, and efforts geared to improving sodium levels during this time, which is associated with better short-term outcomes [16]. In the terminal stages of heart failure, hyponatraemia becomes refractory and is often indicative of severe multi-organ dysfunction. The duration of this phase is variable, but hyponatraemia is typically persistent until death.

Hyponatraemia at this stage is thus driven by hormone mechanisms, which have been triggered by the underlying cardiac process. The question of whether a cardiologist, endocrinologist, or geriatrician is best suited to manage hyponatraemia in heart failure is complex and depends on several factors, including the patient's overall clinical status and comorbidities and the specific expertise required at different stages of management.

Management of hyponatraemia

Research has demonstrated that correcting hyponatraemia in heart failure patients can lead to better clinical outcomes. Treatment of hyponatraemia can alleviate symptoms such as fatigue, confusion, and nausea, which are common in patients with heart failure.

Hyponatraemia has also been found to be a predictor of all-cause mortality in patients with HFpEF in the Karolinska-Rennes (KaRen) study, in which hyponatraemia was a predictor of all-cause mortality with a hazard ratio of 2.69 [20].

Effective treatment is associated with reducing acute decompensation episodes and improving hospitalisation outcomes. To improve longevity, it is crucial to integrate the management of hyponatraemia into the broader therapeutic strategy for heart failure. This includes regular monitoring of sodium levels, individualised treatment plans to manage fluid intake and diuretics, and the use of specific medications when appropriate. 

The management of hyponatraemia in heart failure is often interdisciplinary. Cardiologists are ideally the most appropriate primary physicians due to their comprehensive understanding of heart failure and its complications. However, when hyponatraemia is refractory to initial treatment, as it tends to occur commonly, or when an endocrine disorder is suspected, consultation with an endocrinologist is warranted. For older adults with heart failure and multiple comorbidities, a geriatrician's involvement ensures that all aspects of the patient's health are considered. In these patients, hyponatraemia is often associated with polypharmacy, and drug rationalisation is often helpful [23].

The typical scenario

A common scenario involves an elderly patient with longstanding heart failure who undergoes routine blood tests in the community for an unrelated condition, who is then found to have moderate to severe hyponatraemia. Hyponatraemia can also be detected following admission for other causes, including a fall. Hyponatraemia in these settings is likely a marker of poor prognosis of the underlying heart failure, usually heralding long or multiple hospital stays and the consequent deconditioning. In these patients, quality of life should take precedence, as their primary concern is often maximising time spent at home rather than in hospital beds.

While the patient may not have symptoms due to the low sodium levels, the clinician, however, tries to correct this abnormality to safe levels in view of the risks associated with hyponatraemia. This tends to result in prolonged hospital admission that typically lasts 5-7 days on average and even longer while attempting to correct the sodium level using conventional treatments. Unfortunately, these groups of patients are at higher risk for complications associated with hospitalisation, such as infections and pulmonary embolism, which may pose a greater threat to their lives than the hyponatraemia per se.

Heart failure medications, morbidity, and mortality

The treatment of chronic heart failure emphasises the use of medications that improve mortality as well as symptom control. Four medication classes have traditionally been identified to provide benefits in reducing cardiovascular mortality, all-cause hospitalisation, as well as hospitalisations for heart failure. The summary of the evidence suggests that all patients with HFrEF should be started on a combination of angiotensin receptor-neprilysin inhibitors (ARNIs), sodium-glucose cotransporter-2 (SGLT2) inhibitors, beta-blockers, and mineralocorticoid receptor antagonists (MRAs) to achieve optimum benefit. These medications are regarded as the four pillars of heart failure treatment, and guidelines recommend optimal titration to achieve the greatest benefit [24]. The emphasis is on the adoption of timely initiation and titration of guideline-directed medical treatment (GDMT) in all patients.

In patients admitted for acute decompensation of heart failure, the immediate goals are decongestion, optimisation of medication, and early discharge. The ESCAPE (Evaluation Study of Congestive Heart Failure and Pulmonary Artery Catheterization Effectiveness) trial, which followed up 423 patients after hospital discharge for advanced heart failure, identified low serum sodium as a marker of poor prognosis [25].

In two groups of patients with acute heart failure exacerbation, the use of tolvaptan in one group resulted in shorter length of hospital stay, although this was not statistically significant. Patients on tolvaptan had an adjusted mean hospital stay that was 1.72 days shorter than those on placebo [26]. A study in the United States also compared costs among patients with heart failure and hyponatraemia treated with tolvaptan versus fluid restriction based on the EVEREST trial [27]. There was a potential cost saving of $265 in patients treated with tolvaptan. 

In the AQUA-AHF (Aquaresis Utility for Hyponatremic Acute Heart Failure) trial [28], the use of tolvaptan resulted in similar diuresis when compared with intravenous furosemide in patients who presented in acute decompensated heart failure. In addition, there was no superior diuresis with tolvaptan. Serum sodium level did not differ between the groups on tolvaptan and those on furosemide at the beginning of the trial. However, at the end of the trial, patients on tolvaptan, as expected, had improvement with an increase in sodium levels, while the sodium levels decreased in patients in the furosemide arm. The National Health Service (NHS) England has recommended tolvaptan for hyponatraemia in patients with cancer awaiting chemotherapy and poor response to fluid restriction [29]. Hence, the use of tolvaptan in the NHS is restricted to hyponatraemia due to SIADH in the setting of cancer awaiting chemotherapy [30]. Tolvaptan is not recommended by the National Institute for Health and Care Excellence (NICE) for heart failure or hypovolaemic hyponatraemia. Tolvaptan was approved by the Food and Drug Administration (FDA) in 2009 for the treatment of hyponatraemia in heart failure as well as other hypervolaemic and euvolaemic states. A recent update published in 2017 now limits its use to 30 days and has also advised against its use in patients with liver damage; hence, it is no longer recommended for hyponatraemia due to liver cirrhosis [21].

The role of tolvaptan in managing hyponatraemia in heart failure

Tolvaptan is a selective vasopressin V2 receptor antagonist that has been used for the management of hyponatraemia, particularly in cases associated with heart failure and SIADH. The drug works by inhibiting the action of vasopressin on renal collecting ducts, promoting free water excretion without significant sodium loss, thereby increasing serum sodium levels. Traditional methods of managing heart failure, some of which contribute to the development or worsening of hyponatraemia, include medication and treatment that may themselves worsen heart failure. Although other heart failure medications, such as ANRIs, MRAs, and SGLT2 inhibitors, have modest effects on raising sodium levels, tolvaptan has been shown to have a consistent effect on improving hyponatraemia. While other interventions could alleviate heart failure symptoms, some can themselves exacerbate heart failure, including the four pillars of heart failure management.

The economics of heart failure are enormous. Heart failure is estimated to cost the NHS about £2bn annually, one million inpatient bed days, and 5% of all emergency medical admissions. The length of hospital stay is estimated to be 6-9 days, though this depends on the need for or availability of specialty cardiology input [31]. Tolvaptan is used for managing heart failure-associated hyponatraemia in the United States and some European countries. Its use is more prevalent in these regions due to the drug's approval and availability, as well as its incorporation into clinical practice guidelines for specific indications related to hyponatraemia. However, in the United Kingdom, the use of tolvaptan for heart failure-associated hyponatraemia is limited by several factors, including regulatory and licensing restrictions from NICE as it is not currently approved in acute heart failure [32]. There also remain concerns about cost-effectiveness and the benefit-risk profile, ongoing debates about its overall clinical benefit and cost-effectiveness [33], limited long-term clinical evidence such as mortality and hospitalisation rates, and safety concerns such as liver toxicity, which can be challenging to monitor in routine clinical practice. This includes regular liver function tests and the careful monitoring of sodium levels to avoid complications [3].

Despite tolvaptan's effectiveness in correcting hyponatraemia, it has not found favour with cardiologists as a common agent for the management of hyponatraemia in this group of patients in many parts of the world. Clinical trials such as the EVEREST trial have shown that it does not significantly reduce mortality or the risk of heart failure-related hospitalisations [7], and liver toxicity has led to the recommendation that it be used only in specific situations and under close monitoring. The cost and accessibility issues, as it is expensive, may limit its availability for many patients, especially in resource-constrained settings. This cost-benefit issue further complicates its use in chronic heart failure management, where long-term treatment is often necessary. 

Tolvaptan is recommended for use in hyponatraemia associated with heart failure primarily due to its ability to specifically target the underlying pathophysiological mechanisms that lead to this electrolyte imbalance. Tolvaptan is a vasopressin V2 receptor antagonist that works by promoting aquaresis, which is the excretion of free water without significant loss of electrolytes like sodium. This helps correct dilutional hyponatraemia, a common complication in heart failure patients, without exacerbating electrolyte imbalance [16].

Tolvaptan has been shown to effectively raise serum sodium levels in patients with heart failure. This is particularly important because hyponatraemia is a marker of poor prognosis in heart failure, associated with increased mortality and morbidity [2]. By correcting hyponatraemia, tolvaptan can potentially improve outcomes, although its impact on long-term mortality is still debated.

Hyponatraemia in heart failure patients is often associated with symptoms such as confusion, fatigue, and headaches. By correcting serum sodium levels, improving congestion and renal function, tolvaptan may alleviate these symptoms and consequently improve the overall quality of life for these patients [34]. When compared to traditional therapies like fluid restriction, which can be difficult to maintain and are often ineffective, tolvaptan provides a targeted approach with a manageable safety profile when used in the short term. This can be particularly beneficial in acute settings, and care has to be taken with the rapid correction of sodium levels [35].

Clinical studies on tolvaptan in hyponatraemia and heart failure

There have been several studies on the use of tolvaptan in heart failure and patient outcomes. In the EVEREST trial [7], patients randomised to tolvaptan achieved an improvement in their sodium from baseline by 5.49 meq/L at day 7 or discharge, compared to 1.85 meq/L in the placebo group. However, the trial was limited by the fact that only 8% of the patients had an initial sodium level less than 134 meq/L. There was no reported benefit in mortality or morbidity among the patients on tolvaptan; however, the patients on tolvaptan lost more weight and had better improvement of their dyspnoea. Patients with hyponatraemia on tolvaptan also received fewer doses of diuretics and were more likely to normalise their sodium levels prior to discharge.

The SECRET of CHF (Study to Evaluate Challenging Responses to Therapy in Congestive Heart Failure) trial evaluated the effect of the addition of tolvaptan to diuretic therapy on the alleviation of dyspnoea in patients with acute heart failure [19]. This was a double-blind study in patients who had hyponatraemia. They were randomised to receive either 30 mg daily of tolvaptan or a placebo. Self-assessed dyspnoea was recorded using a 7-point scale at eight and 16 hours following admission. There was no significant difference in perceived dyspnoea between the tolvaptan arm and the placebo arm. The patients on tolvaptan required lower doses of diuretics than those on placebo; however, this was not statistically significant. The patients on tolvaptan also showed a tendency toward higher sodium levels, and this was also not statistically significant. However, the number of patients was small, with only 41 patients documented to have hyponatraemia at baseline. This small sample size means the trial was underpowered; hence, results should be interpreted with caution.

Another major trial of tolvaptan in heart failure was the TACTICS-HF (Targeting Acute Congestion with Tolvaptan in Congestive Heart Failure) study [36]. This was conducted to address the acute use of tolvaptan to improve congestion in acute decompensated heart failure. In the trial, tolvaptan was associated with greater fluid loss and weight loss than placebo; however, there was no difference in dyspnoea, need for rescue therapy, or length of hospital stay between the two groups. The trials above were essentially similar in terms of the dose of tolvaptan used in the patients. In the studies mentioned above, 30 mg once a day of tolvaptan was used either daily until discharge in EVEREST or as three STAT doses at 0, 24, and 48 hours in TACTICS-HF and seven days in SECRET of CHF, respectively.

The SALT (Study of Ascending Levels of Tolvaptan in Hyponatraemia) trials were major trials on the use of tolvaptan for the management of hyponatraemia [37,38]. The SALT-1 and SALT-2 trials were randomised, double-blind placebo trials in both euvolaemic and hypovolaemic hyponatraemia. Patients in the trial had either heart failure, cirrhosis, or SIADH. These patients were given either 15 mg/day of tolvaptan or a matching placebo for 30 days. Tolvaptan dose was adjusted during the study to maintain sodium between 135 and 145 meq/L. The effect of tolvaptan in hyponatraemia was noticed at eight hours. In the SALT-1 trial, hyponatraemia was due to heart failure in 33% of recruited patients, while heart failure-associated hyponatraemia was present in 29% in SALT-2. This study was performed in an outpatient setting. The two studies demonstrated that tolvaptan was superior to placebo in improving sodium levels despite the severity of hyponatraemia at baseline. 

Heart failure treatment has evolved over the years, with emphasis shifting beyond symptomatic relief to the use of medication with proven survival benefits. These medications include angiotensin-converting enzyme (ACE) inhibitors, beta-blockers, ARNIs, MRAs, and SGLT2 inhibitors. Tolvaptan has not been shown to have a beneficial effect on mortality so far in studies. Although hyponatraemia has been shown to be a predictor of poor prognosis in heart failure, there is no clear evidence that tackling this in isolation improves mortality. However, one can infer that correcting hyponatraemia is expected to shorten hospital stay and improve symptoms directly attributable to hyponatraemia, such as falls and confusion. Nonetheless, it has yet to be proven that this can improve quality of life, and more studies are needed.

Table 1 summarises some of the trials that have been done to evaluate the use of tolvaptan in hyponatraemia in heart failure.

**Table 1:** A comparison of major studies on use of tolvaptan in heart failure EVEREST: Efficacy of Vasopressin Antagonism in Heart Failure: Outcome Study with Tolvaptan; SALT: Study of Ascending Levels of Tolvaptan in Hyponatraemia; AQUA-AHF: Aquaresis Utility for Hyponatremic Acute Heart Failure; SECRET of CHF: Study to Evaluate Challenging Responses to Therapy in Congestive Heart Failure; TACTICS-HF: Targeting Acute Congestion with Tolvaptan in Congestive Heart Failure; SIADH: syndrome of inappropriate antidiuretic hormone secretion

Study	Patient population	Setting	Outcome in patients on tolvaptan
EVEREST	Acute heart failure	Inpatient	Improved sodium levels, reduced diuretic doses, no impact on mortality and hospitalisation
SALT	Chronic heart failure, cirrhosis, SIADH	Outpatient	Improved sodium levels
AQUA-AHF	Acute heart failure	Inpatient	Similar diuresis compared with intravenous furosemide; serum sodium increased.
SECRET of CHF	Acute heart failure with serum sodium <135 mmol/l; small sample size	Inpatient	No early reduction in dyspnoea; better weight reduction and less diuretic dose
TACTICS-HF	Acute heart failure	Inpatient	No difference in length of hospital stay and clinical benefit, including symptoms; however, improved weight loss and fluid loss

Cost implications of tolvaptan compared to the conventional and current treatments

One of the major drawbacks to the use of tolvaptan is its high cost. Although tolvaptan is more expensive per dose than traditional therapies, its ability to reduce hospital stays and associated complications may make it a cost-effective option in elderly patients with severe hyponatraemia. The financial benefits, coupled with the potential to improve quality of life and mitigate hospital-related risks, highlight the value of this targeted therapy in carefully selected cases. The cost of a single 30 mg dose of tolvaptan is approximately £43.15 (based on a seven-tablet pack priced at £302.05). While this may appear expensive compared to traditional diuretics such as furosemide (28 tablets for £0.67) [39], the broader economic implications reveal a more favourable outlook for tolvaptan in certain scenarios. However, there may be hidden costs with its use, such as monitoring liver function and sodium, and these will need to be considered in the health economic calculations.

In the studies cited above, the use of tolvaptan for acute heart failure was done with a maximum of a seven-day course of 30 mg tablets. The effects on hyponatraemia were inconsistent at best due to the small number of patients enrolled in the trial.

Chiong et al. carried out a cost analysis based on the EVEREST trial on the use of tolvaptan in heart failure. They identified a shorter length of hospital stay in tolvaptan-treated patients than placebo (9.72 vs. 11.44 days, respectively), leading to an estimated cost saving of $265 per patient per admission [37].

A similar analysis of data obtained from the SALT-1 and SALT-2 trials also demonstrated that the use of tolvaptan in patients with SIADH was associated with a shorter hospital length of stay and cost reduction of $694 per admission [40].

Prolonged hospitalisations, often necessary to correct severe hyponatraemia in elderly patients with heart failure, are associated with substantial healthcare costs. These include inpatient care, nursing support, diagnostic investigations, and treatment for complications like hospital-acquired infections, delirium, and thromboembolic events.

Hospital Bed Costs

Delayed discharges from hospitals create an enormous financial burden for the NHS. The charity Age UK estimated that delayed discharges from hospital to social care in the community would cost the NHS £587m (€695m; $770m) in the period from June 8, 2017, to December 12, 2019 [41].

Complication Costs

Managing complications such as hospital-acquired infections can increase costs significantly. Bed days lost to hospital-acquired infections in Scotland during 2017/2018 were estimated to cost £46.4 million [42].

Indirect Costs

Prolonged hospital stays can adversely affect elderly patients' quality of life and increase caregiver burden, although these are harder to quantify financially [42].

When tolvaptan is used to rapidly correct serum sodium levels, e.g., in EVEREST [27] and SALT [37] trials, it has shown a potential reduction in hospital length of stay by 1-2 days on average. Even modest reductions in hospitalisation duration could offset the immediate costs of tolvaptan. Additionally, by preventing complications associated with prolonged hospital stays, further cost savings and improved patient outcomes may be achieved.

Limitations

Although lots of studies have been done on the role of tolvaptan in heart failure, these have been relatively small trials with short-term follow-up. Conflicting results of the trials, with no clear benefits of tolvaptan on mortality and rehospitalisation rates, as well as an increase in side effects, appear to have largely limited the wide acceptability of using tolvaptan for this purpose. 

## Conclusions

Hyponatraemia in heart failure is not merely a marker of disease severity but is associated with adverse outcomes, particularly in elderly patients with advanced heart failure. The typical scenario of a frail, elderly patient with heart failure and severe hyponatraemia who ends up in a prolonged hospital stay with its attendant risks is common. The use of tolvaptan could play a role in mitigating this.

This narrative review, therefore, highlights the potential of tolvaptan to address a critical unmet need: correcting serum sodium levels, reducing clinical congestion without significant side effects, and reducing the length of hospital stay. It is, however, unlikely to have a mortality benefit; hence, this must be taken into consideration and requires an individual approach to its use. While the benefits of treating heart failure with tolvaptan on mortality remain unproven, our findings suggest that tolvaptan can meaningfully improve clinical outcomes by facilitating earlier discharge and reduced inpatient costs, mitigating hospital-related complications, and enhancing symptom management. However, the limitations of previous studies have been highlighted, and more robust studies could help to clarify whether tolvaptan can become a preferred treatment in this group of patients.
